# Role of Immunotherapy in Gastroesophageal Cancers—Advances, Challenges and Future Strategies

**DOI:** 10.3390/cancers15225401

**Published:** 2023-11-14

**Authors:** Emer Lynch, Austin G. Duffy, Ronan J. Kelly

**Affiliations:** 1 Department of Medical Oncology, The Mater Hospital, D07 R2WY Dublin, Ireland; emeralynch@gmail.com (E.L.); austinduffy@mater.ie (A.G.D.); 2 The Charles A. Sammons Cancer Center, Baylor University Medical Center, Dallas, TX 75246, USA

**Keywords:** gastric cancer, esophageal cancer, gastroesophageal cancer, immunotherapy, immune checkpoint inhibitors

## Abstract

**Simple Summary:**

Gastric and esophageal cancers represent a global health concern with considerable associated morbidity and mortality. The management of these malignancies was historically limited to chemotherapy, radiotherapy and surgery. More recently, immunotherapies, which can harness the immune system to treat cancer, have become an established treatment option in many cancer types. This article presents the existing evidence for the use of immunotherapies in gastric and esophageal cancers that are localized to one area or that have spread to other areas. It considers challenges in terms of the use of these agents and also discusses immune-based treatments that are currently under investigation in this area which may potentially change treatment practices in the future.

**Abstract:**

Background: Gastroesophageal cancers (GECs) carry considerable morbidity and mortality, and demonstrate geographical histological variances in addition to molecular heterogeneity. Consequently, the immunogenicity of the different subtypes, which can predict the likelihood of immunotherapy response, can vary. Immune checkpoint inhibitor (ICI) therapy has transformed the treatment of many cancer types over the past decade but has been slower to gain a foothold in the treatment paradigm of GECs. Methods: This article reviews the existing evidence and use approvals for immunotherapies and immune-based treatments in GECs, in the neoadjuvant, adjuvant and metastatic disease settings. The challenges of and limitations to ICI application in current clinical practice are examined. Ongoing clinical trials and future directions of research are also considered. Conclusion: ICI therapy has become an established treatment option within GECs, both perioperatively and in advanced disease. However, nuances in terms of its use are not yet fully understood. Ongoing research proposes to broaden the application of immunotherapies in GECs with the potential to continue to improve outcomes.

## 1. Introduction

Internationally, gastric and esophageal cancers represent the fifth and seventh most common cancers, respectively, each with considerable associated mortality [1]. Significant geographical variations exist in the incidence of the different histological and topographical subtypes, largely related to their underlying etiological causes. In Western countries, trends are towards rising cases of distal esophageal and junctional adenocarcinomas. This is largely due to decreasing rates of chronic *Helicobacter pylori* infection and rising incidence of obesity, gastroesophageal reflux disease and Barrett’s metaplasia [2]. Gastric cancer incidence in the West is decreasing and is often classified by topographical subtype, into cardia and non-cardia. It is important to consider such etiological, anatomical and histological heterogeneity when evaluating the immunogenicity of gastroesophageal malignancies. The United States (U.S.) Food and Drug Administration (FDA) and European Medicines Agency (EMA) approved indications for immune checkpoint inhibitors in gastroesophageal cancers (GECs) have differing biomarker and histology requirements depending on the anatomic site of origin. A minority of patients derive significant benefit from programmed cell death protein 1 (PD-1) inhibition, so the use of this class of drugs in upper gastrointestinal (GI) tumors requires a nuanced understanding of who is likely to benefit. At the time of writing, microsatellite instability high (MSI-H) and mismatch repair deficient (dMMR) status and the programmed death-ligand 1 (PD-L1) combined positive score (CPS) remain the most useful predictive biomarkers, but it is hoped that ongoing work evaluating gene expression signature scores and a more comprehensive understanding of the immune microenvironment will help treating oncologists make informed choices in the years ahead.

## 2. Immunogenicity of Gastroesophageal Cancer

In addition to histological classification, the Cancer Genome Atlas proposed a molecular based classification, in order to more accurately characterize and manage gastric adenocarcinomas, and this includes four separate subtypes. Of these, two subtypes, Epstein Barr Virus (EBV) infected and microsatellite unstable (MSI) tumors, account for 9% and 22% of gastric cancers, respectively, and can be characterized as ‘immunogenic’ with noted upregulation of immune-related genetic pathways [3]. Both subtypes have demonstrated excellent responses to immunotherapy, with evidence in MSI tumors for both single agent immune checkpoint inhibitors and in combination with chemotherapy over chemotherapy alone [4,5,6]. Similarly, in EBV-positive gastric cancer, objective response rates (ORRs) of up to 100% have been seen with immune checkpoint inhibition, albeit from small datasets [7]. In contrast, molecular subtyping of esophageal squamous cell carcinoma (ESCC) has failed to deliver any specific subsets that can determine likelihood of response to immune checkpoint inhibitors (ICIs) [8]; however, as will be discussed, there is evidence of broad response to the ICI class in ESCC with a number of positive phase III studies in this setting. In particular, ESCCs have been found to demonstrate higher levels of PD-L1 and neoantigen expression compared to esophageal adenocarcinoma (EAC) [9,10,11].

However, even outside of the MSI-H- and EBV subtypes which infer considerable immunogenicity by subtype alone, the immune response or lack thereof can be shown to play a role in the proliferation of gastroesophageal tumors. Malignant cells can be seen to evade the adaptive and innate immune system by a number of mechanisms including down-regulating antigens and major histocompatibility complexes, directly suppressing the immune response using tumor-associated macrophages and inducing T cell inactivation [12]. Malignancies can typically be characterized as T-cell inflamed or not, dependent on the extent of infiltration of immune cells, with the degree of key inflammatory tumor-infiltrating lymphocytes (TILs) important to predict the degree of immune response [13,14]. Such factors can be further influenced by therapeutics, with chemoradiation evidenced to increase PD-L1 expression and certain chemotherapies including oxaliplatin proving more immunogenic than others [15,16]. These factors are important to consider, as the role for combined chemoimmunotherapy and multi-modality treatment has become established within the GEC setting.

Furthermore, there is evidence that the degree of PD-L1 expression in GEC can indicate likelihood of response to ICI therapy, albeit with considerable limitations [17]. It is acknowledged there can be substantial heterogeneity in PD-L1 expression within a single tumor, in addition to differences in PD-L1 expression between a primary tumor and its secondary metastases [18]. For these reasons, while PD-L1 expression has evolved into one of the key biomarkers used in GEC, it remains an imperfect tool. In particular, it should be recognized there exists considerable variation across clinical trials regarding which PD-L1 assay was used, and whether a combined positive score (CPS) or a tumor proportion score (TPS) was used, and this information is relevant in interpreting trial results.

## 3. Immunotherapy in Early Gastroesophageal Cancer

In the early disease setting, over the past two decades, perioperative treatment with chemotherapy alone or chemoradiotherapy has become an established standard of care in GEC. Neoadjuvant chemoradiotherapy as per the CROSS regimen has been shown to deliver pathological complete response (pCR) rates of almost 30% [19], with pCR acknowledged as a surrogate marker for improved overall survival (OS) in the perioperative setting [20]. Similarly, the FLOT chemotherapy regimen (docetaxel, oxaliplatin and fluorouracil) administered pre- and post-operatively demonstrates improved OS results in comparison with earlier, more toxic regimens such as ECF (epirubicin, cisplatin and 5-fluorouracil), in gastric or gastroesophageal junction (GEJ) adenocarcinomas [21]. However, there is considerable scope for improvement in both pCR and OS rates in the perioperative setting, and ICI therapy has already begun to transform the treatment paradigm in early-stage disease.

The CheckMate 577 trial evaluated adjuvant ICI therapy in patients with resected stage II and III esophageal and GEJ cancers post neoadjuvant chemoradiotherapy who had residual pathological disease post-operatively [22]. Patients were randomized 2:1 to either nivolumab or placebo to complete a year of treatment. Of note, 71% of patients had the adenocarcinoma histologic subtype and 29% had squamous cell. At a median follow-up of 24.4 months, median disease-free survival (DFS) was 22.4 months in the nivolumab group compared to 11 months in the placebo arm (HR 0.69; 96.4% CI, 0.56 to 0.86; *p* < 0.001). Adjuvant nivolumab has FDA and EMA approval in this setting since 2021.

Building on this, there are a number of immunotherapy studies currently underway or that have recently been presented within the perioperative setting albeit with results not yet proving practice-changing thus far. EORTC 1707 Vestige is a European open label phase II trial assessing adjuvant nivolumab 3 mg/kg every 2 weeks with Ipilimumab 1 mg/kg every 6 weeks for one year versus standard adjuvant chemotherapy using the same regimen as received pre-operatively for patients with gastroesophageal adenocarcinoma [23]. All patients received neoadjuvant chemotherapy and had to have node positive and/or R1 resection status for inclusion. At a planned interim analysis at median follow-up of 11.1 months, median DFS was 23.3 months in the chemotherapy arm in comparison with 11.9 months in the combined ICI arm (95% CI 1.09–2.98, *p* = 0.02), and median OS in those who received chemotherapy was not reached versus median OS of 25.1 months for those who received combined ICI (HR 1.79, 95% CI 0.89–3.59, *p* = 0.1). Based on these results, the independent data monitor advised further enrollment to the trial should cease due to a lack of activity. Subgroup analyses, in particular looking at PD-L1 and dMMR/MSI-H, are awaited.

In a similar vein, but looking at the intrinsically immunotherapy-sensitive subgroup of deficient mismatch repair and MSI-H patients, GERCOR NEONIPIGA looks to omit chemotherapy entirely in the perioperative setting [5]. In an open label phase II trial, 32 patients were enrolled with dMMR and MSI-H locally advanced but resectable T2–T4 NxM0 gastric and GEJ adenocarcinoma to receive neoadjuvant nivolumab 240 mg every 2 weeks for 6 cycles with ipilimumab 1 mg/kg every 6 weeks for 2 cycles, before proceeding to surgery and adjuvant nivolumab 480 mg every 4 weeks for 9 cycles. Primary endpoint was pCR. At a median follow-up of 14.9 months, 27 patients had completed all neoadjuvant immunotherapy, and 29 patients proceeded to surgery. Of the three that did not, two declined surgery and one had had metastatic disease at inclusion, and all three had complete response noted on endoscopic assessment with normal imaging. Of the 29 patients who proceeded to surgery, all had an R0 resection and 17 (58.6%) had pCR, with 23 patients proceeding to adjuvant nivolumab. One post-operative death occurred. At data lockout of just over one year, no patient had yet relapsed.

INFINITY, a phase II single-arm trial, also looked at the role of combined ICI therapy in the early disease setting in this same cohort and was presented at the American Society of Clinical Oncology (ASCO) GI symposium 2023 [24]. Patients with MSI-H/dMMR and EBV negative gastric and gastroesophageal junction adenocarcinoma were recruited to two cohorts, to look at combined tremelimumab targeting cytotoxic T-lymphocyte-associated protein 4 (anti-CTLA-4) and durvalumab (anti-PD-1) in the neoadjuvant setting for cohort 1 and as definitive management for cohort 2. Eighteen patients were recruited to cohort 1, with a primary endpoint of pCR, and received tremelimumab 300 mg with durvalumab 1500 mg every three weeks for four cycles. One patient withdrew their consent, two patients declined surgery having achieved radiological and endoscopic pCR, and one patient had progressive disease. Of the 14 that proceeded to surgery, a pCR rate of 60% was seen. Higher pCR rates were seen in patients with T2/T3 tumors (89%) than in those with T4 disease (17%). Grade 3 or higher AEs were seen in three patients. Enrollment to the second cohort is underway.

Impressive improvements have been seen with the addition of immunotherapy to chemotherapy in the neoadjuvant setting in other primary malignancies, including triple negative breast cancer [25]. Given the survival advantage provided with perioperative FLOT chemotherapy, a similar rationale underlies the KEYNOTE-585 phase III trial which investigates the addition of pembrolizumab to chemotherapy in the perioperative setting, in patients with operable T3 or greater or node positive gastric and GEJ adenocarcinoma [26]. Results were presented as a late breaking abstract at the European Society for Medical Oncology (ESMO) Congress in October 2023 [27]. Patients received three cycles of chemotherapy (cisplatin and 5-Fu or capecitabine) every three weeks with pembrolizumab or placebo, for three cycles pre-operatively and three cycles post-operatively, followed by up to 11 cycles of pembrolizumab or placebo, with a separate cohort assessing perioperative FLOT, current standard of care with the addition of pembrolizumab or placebo. N = 804 patients were randomized in the main cohort and N = 203 in the FLOT cohort. At median follow-up of 47.7 months, over 90% of patients completed neoadjuvant treatment, 82–88% of patients completed surgery with surgery completion rates of 87% and 88% in the FLOT arms, and about half of patients completed all adjuvant therapy. In the main cohort, greater pCR rates were seen with the addition of pembrolizumab with a 12.9% pCR rate in the immunotherapy arm versus 2% in the placebo arm (treatment difference of 10.9% [95% CI, 7.5–14.8]; *p* < 0.00001). Similar results were seen when the results for the FLOT cohort were included with the main cohort, with an overall pCR rate of 13% for chemotherapy with immunotherapy and 2.4% for chemotherapy alone (10.6% treatment difference [95% CI, 7.4–14], *p* < 0.0001). Results for the FLOT cohort were not independently presented. Event-free survival results were not statistically significant, with a median of 44.4 months with immunotherapy versus 25.3 months with placebo, but HR 0.81 (95% CI, 0.67–0.99). Of note, the trial accepted all-comers, regardless of PD-L1 status. Within the stratification, improved hazard ratios were seen to favor the addition of immunotherapy in the PD-L1 CPS ≥ 10 [0.70 (0.46–1.04)] and for the MSI-H cohort [0.59 (0.24–1.47)]. Overall survival data are as yet immature, and grade 3–4 adverse events were similar across both pembrolizumab and placebo arms. While these results are somewhat disappointing, they must be considered against the limited number of patients who received standard of care FLOT, and the existing evidence for a synergistic effect between immunotherapies and oxaliplatin, with oxaliplatin recognized as an immunogenic cell death agent, whereas cisplatin is not [28,29].

Interim results from MATTERHORN, looking at the addition of immunotherapy to perioperative FLOT, were also presented at ESMO 2023 [30]. A global phase III trial, it randomized patients with resectable stage II or III gastric or gastroesophageal junction adenocarcinoma to either durvalumab 1500 mg or placebo every four weeks with FLOT every two weeks on days 1 and 15, to complete 2 cycles pre-operatively and two cycles post-operatively [31]. N = 948 patients were randomized, with 90% of patients having T3 or T4 disease, and almost 70% of patients were lymph-node positive. In each arm, a similar percentage proceeded to surgery (87% with durvalumab vs. 84% with placebo) and continued with adjuvant treatment (45% immunotherapy arm and 43% placebo arm). Centrally assessed pCR rates were 19% in the durvalumab arm and 7% in the placebo arm, a difference of 12% (odds ratio 3.08; *p* < 00001), with results consistent across all subgroups. The addition of durvalumab was well tolerated, and the study is ongoing for its primary endpoint of event-free survival (EFS). Given the absence of EFS data for MATTERHORN, and the failure to deliver a statistically significant improvement in EFS in KEYNOTE-585, there is insufficient evidence at the current time to support the routine use of ICI therapy in the perioperative setting.

Finally, results of the phase IIb DANTE trial were presented at ASCO 2022 [32]. Again, using the FLOT backbone, it randomized patients with resectable gastric or GEJ adenocarcinoma with clinical tumor staging of ≥T2 or node positive disease to complete 4 cycles neoadjuvant and 4 cycles adjuvant chemotherapy with or without atezolizumab 840 mg every 2 weeks for 8 cycles, followed by atezolizumab 1200 mg every 3 weeks for 8 cycles, with a primary endpoint of progression-free survival (PFS). N = 295 patients were randomized, with 8.5% MSI-H, and 50% demonstrating PD-L1 CPS of ≥1, 23% PD-L1 CPS ≥ 5, and 15% expressing PD-L1 CPS ≥ 10. Just over 90% in both arms achieved an R0 resection with similar surgical morbidity, and no difference between the cohorts in terms of perioperative chemotherapy completion rates. Greater tumor downsizing was seen in the immunotherapy arm, with pT0 results seen for 23% in comparison with 15% for chemotherapy alone, and pN0 in 68% with ICI versus 54% in the chemotherapy arm. Improved tumor regression rates were also achieved with the addition of ICI to chemotherapy, with greater rates observed with the higher PD-L1 CPS scores and for those who were MSI-H. Although pCR rates were similar in both arms overall, for those with PD-L1 CPS ≥ 10, centrally assessed pCR was 46% in the atezolizumab arm and only 24% for those receiving chemotherapy alone. DANTE continues to accrue as a phase III trial, although based on the subgroup analyses from the phase II data, enrollment is now limited to those with MSI-H, PD-L1 CPS of ≥ 10, tumor mutational burden (TMB) of ≥ 10/MB and those who are EBV positive [33]. Ultimately, albeit with some phase III survival data yet outstanding, it may prove that the role of perioperative chemoimmunotherapy in gastric/GEJ adenocarcinoma may be limited to those with immunologically hot tumors, as has largely been seen in the advanced disease setting. Table 1 summarizes relevant active and resulted trials in the peri-operative setting and their associated FDA and EMA approvals. 

## 4. Advanced/Metastatic HER2-Negative—First-Line Treatment

The earliest evidence for the efficacy of immunotherapy in GEC was seen with single agent pembrolizumab in the advanced disease setting. Early-phase clinical trials KEYNOTE-012 and KEYNOTE-028 demonstrated ORRs of 22% and 30% in PD-L1 positive pre-treated gastric and esophageal cancers, respectively [34,35]. Building on this, KEYNOTE-059 enrolled as a non-randomized phase II trial assessing pembrolizumab monotherapy in advanced gastric or GEJ adenocarcinoma with at least two previous lines of treatment [36]. ORR was 11.6%, and was marginally higher in those who were PD-L1 positive (PDL-1 ≥ 1) at 15.5% (95% CI, 10.1–22.4%) in comparison with 6.4% in the PD-L1 negative cohort. As a result, pembrolizumab monotherapy was approved in this setting by the U.S. Food and Drug Administration (FDA) in September 2017. However, this indication was subsequently withdrawn in 2021 following a recommendation by the FDA’s Oncologic Drugs Advisory Committee (ODAC), based on the subsequent results of KEYNOTE-061 and KEYNOTE-062, where overall survival criteria were not met [37,38].

KEYNOTE-590 established a new standard of care for combined chemotherapy with immunotherapy in the first-line setting for patients with advanced disease [39]. A phase III randomized control trial, it evaluated pembrolizumab or placebo in combination with chemotherapy (5- fluorouracil and cisplatin) in patients with advanced esophageal cancer or Siewert type 1 gastroesophageal junction cancer (GEJC). N = 749 patients were enrolled; a majority were histological subtype ESCC (73%), and 27% had adenocarcinoma histology with 12% of the total cohort enrolled with Siewert type 1 GEJC adenocarcinoma. A first interim analysis at median follow-up of 22.6 months demonstrated improved OS with combined chemotherapy and ICI of 12.4 months versus 9.8 months with chemotherapy alone (HR 0.73 [95% CI 0.62–0.86]; *p* < 0.0001). Greater benefits were seen with the addition of immunotherapy for those with both PD-L1 CPS ≥ 10 and ESCC histology where OS was 13.9 months versus 8.8 months with chemotherapy alone (HR 0.57 [95% CI 0.43–0.75]; *p* < 0.0001). Further updated efficacy, safety and quality of life results with an additional 12 months of data were presented at the American Society of Clinical Oncology (ASCO) GI Symposium in 2022 [40]. These revealed continued OS advantage regardless of histopathological subtype, although the benefit of pembrolizumab is likely driven by those with high PD-L1 expression. Based on these data, the FDA approved this regimen for esophageal adenocarcinoma and ESCC, and Siewert Class 1 GEJC. The EMA, however, afforded approval only for those with PD-L1 CPS ≥ 10. As the role of adjuvant nivolumab has been established in ESCC as per CHECKMATE-577 [22], there are questions regarding when to re-challenge with immunotherapy for those with relapsed disease. The general consensus at the current time is to consider ICI therapy if at least 6 months has elapsed since prior adjuvant immunotherapy.

As no gastric tumors were enrolled in KEYNOTE-590, pembrolizumab with chemotherapy was not initially licensed in the advanced gastric and GEJ adenocarcinoma setting. Addressing part of this gap, CHECKMATE 649 was a three-armed trial looking at first-line treatment for advanced gastric, GEJ and esophageal adenocarcinoma [41]. Patients were randomized to either nivolumab plus chemotherapy [CAPOX (capecitabine with oxaliplatin) every 3 weeks or FOLFOX (5-Fluourouracil with oxaliplatin) every 2 weeks], nivolumab plus ipilimumab or chemotherapy alone. Although enrollment to the ipilimumab and nivolumab arm was later closed due to excessive toxicity, it continued for the alternate two arms. N = 1581 patients were randomized to either nivolumab with chemotherapy or chemotherapy alone arms, and about 60% had PD-L1 CPS ≥ 5. Dual primary endpoints of OS and PFS were both met. At median follow-up of 13.1 months for nivolumab with chemotherapy and 11.1 months for chemotherapy alone, the combined chemotherapy with ICI arm showed improved OS of 14.4 months versus 11.1 months for patients with PD-L1 CPS ≥ 5 [HR 0.71, 95% CI 0.59–0.86, *p* < 0.0001). The results were less convincing for all-comers, with median OS of 13.8 months for the nivolumab with chemotherapy in comparison with 11.6 months for chemotherapy alone [HR 0.80, (99.3% CI 0.68–0.94), *p* = 0.0002]. Median PFS for nivolumab with chemotherapy in those with PD-L1 CPS ≥5 [42] was 7.7 months, and 6.05 months for chemotherapy alone [HR 0.68, 98% CI 0.56–0.81, *p* < 0.0001]. Swift FDA approval for the regimen followed in April 2021, regardless of PD-L1 expression, with EMA approval following in September 2021, but only for those with PD-L1 CPS ≥ 5.

Results from the truncated ipilimumab and nivolumab arm from CHECKMATE 649 were later published, along with updated survival data from the nivolumab and chemotherapy and chemotherapy alone cohorts [43]. It is worth noting that the combined immunotherapy arm used a dosing schedule of ipilimumab 3 mg/kg and nivolumab 1 mg/kg, which, although in other malignancies, has been found to demonstrate improved response rates, is associated with a greater level of adverse events (AEs) than when administered as ipilimumab 1 mg/kg and nivolumab 3 mg/kg [42]. At minimum follow-up of 24 months, the OS gains seen for nivolumab with chemotherapy versus chemotherapy alone were sustained; however, the secondary endpoint of OS for ipilimumab with nivolumab versus chemotherapy alone in those with CPS ≥ 5 did not meet statistical significance. Furthermore, although PFS and objective response rate (ORR) were not improved in the combined immunotherapy arm versus chemotherapy for PD-L1 CPS ≥ 5, it is not surprising that nivolumab with ipilimumab delivered more durable responses, for those that did respond, in comparison with chemotherapy for both PD-L1 CPS ≥ 5 (13.2 months vs. 6.9 months, 95% CI 8.3, 18.3; 5.2, 7.6) and all randomized patients (13.8 months vs. 6.8 months, 95% CI 9.4, 17.7; 5.6, 7.2). These data, combined with those from EORTC Vestige in the early disease setting, prompt questions as to whether there is a role for combined ICI therapy with anti-PD-1 and anti-CTLA-4 in gastroesophageal adenocarcinoma.

Perhaps reflecting the known higher levels of inherent PD-L1 expression in ESCC, CHECKMATE 648 has delivered the only potential role, although limited, for combined immunotherapy in the advanced gastroesophageal setting to date [44]. A global phase III trial, CHECKMATE 648 randomized N = 970 patients with previously untreated advanced ESCC on a 1:1:1 ratio to either nivolumab plus chemotherapy (5-fluourouracil plus cisplatin), nivolumab plus ipilimumab or chemotherapy alone. Nivolumab or nivolumab plus ipilimumab were administered for a maximum of two years. Of the patients, 70% were Asian and 49% demonstrated PD-L1 expression of 1% or greater. At minimum follow-up of 13 months, for those with PD-L1 ≥ 1%, median OS was 15.4 months in the chemo-immunotherapy arm and 9.1 months for those receiving chemotherapy alone (HR, 0.54; 99.5% CI, 0.37 to 0.80; *p* < 0.001); and for the entire population it was 13.2 months for nivolumab with chemotherapy versus 10.7 months with chemotherapy alone (HR 0.74; 99.1% CI, 0.58 to 0.96; *p* = 0.002). The trial was not designed to compare the nivolumab plus chemotherapy and nivolumab plus ipilimumab arms directly, rather comparing each separately with chemotherapy alone. For those receiving combined ICI therapy, median OS was 13.7 months in comparison with 9.1 months for chemotherapy (HR, 0.64; 98.6% CI, 0.46 to 0.90; *p* = 0.001) for those with PD-L1 ≥ 1%. For all-comers, improved OS was again noted with nivolumab and ipilimumab with 12.7 months versus 10.7 months (HR, 0.78; 98.2% CI, 0.62 to 0.98; *p* = 0.01). Treatment-related AEs of grade 3 or 4 occurred in 47% patients receiving nivolumab plus chemotherapy, 32% for nivolumab plus ipilimumab and 36% for chemotherapy alone. For those with PD-L1 expression ≤ 1, median OS was similar in all arms at about 12 months; however, for those who did respond, there was a greater percentage experiencing a duration of response greater than 12 months for those receiving either of the nivolumab arms, at 47% for ipilimumab plus nivolumab, 38% for nivolumab plus chemotherapy and 27% for chemotherapy alone. For these reasons, current Society for Immunotherapy of Cancer (SITC) guidelines suggest that for advanced ESCC patients that are chemotherapy ineligible and with PD-L1 TPS ≥ 1, combined ICI therapy with nivolumab and ipilimumab is the first choice therapeutic option [17].

Building on KEYNOTE-590, which, as noted above, enrolled only those with esophageal and GEJ cancers (Siewert 1), KEYNOTE-859 proposed to expand on indications for pembrolizumab with chemotherapy in the advanced upper GI cancers in the front-line setting. Interim analysis results were presented as an ESMO virtual plenary session in February 2023 [45]. Patients with previously untreated advanced gastric and GEJ adenocarcinoma were randomized 1:1 to receive either pembrolizumab or placebo with chemotherapy (either 5-fluorouracil with cisplatin or capecitabine with oxaliplatin, and stratified as per geographical location, PD-L1 status (CPS < 1 or ≥1) and which chemotherapy regimen was received. Of the patients, 78% had PD-L1 CPS ≥ 1, and 35% had PD-L1 CPS ≥ 10. At median follow-up of 31 months, the combined chemotherapy with pembrolizumab arm revealed median OS of 12.9 months versus 11.5 months for chemotherapy alone (HR 0.76, 95% CI 0.67–0.85; *p* < 0.0001), and results were consistent across all subgroups including PD-L1 expression, although again better results were seen for those with greater PD-L1 CPS scores. For PD-L1 CPS ≥ 1, median OS was 13 months for chemo-immunotherapy and 11.4 months chemotherapy alone (HR 0.74, 95% CI 0.65–0.84, *p* < 0.0001) and for CPS ≥ 10, the addition of pembrolizumab demonstrated OS of 15.7 months versus 11.8 months (HR 0.65, 95% CI 0.53–0.79, *p* < 0.0001). Again, the FDA afforded approval for pembrolizumab with chemotherapy for all-comers, in comparison with the EMA approval which is only for those with PD-L1 CPS ≥ 1.

Finally, tislelizumab is a novel anti-PD-1 ICI approved in multiple settings in China that has reported phase III level efficacy in combination with chemotherapy in esophageal cancer. RATIONALE 306, a phase 3 trial within a global population, randomized N = 649 patients with previously untreated advanced ESCC to chemotherapy of investigators’ choice (cisplatin or oxaliplatin with 5-flourouracil or capecitabine or paclitaxel) with tislelizumab or placebo regardless of PD-L1 status [46]. Median OS in the immunotherapy arm was 17.2 months (95% CI 15.8–20.1) in comparison with 10.6 months for chemotherapy alone (stratified HR 0.66 [95% CI 0.54–0.80]; one-sided *p* < 0.0001). Neither EMA nor FDA approval for this combination has been afforded in this setting to date. Table 2 lists resulted and active trials of importance in the first line management of advanced GEC, in addition to current associated FDA and EMA use approvals. 

## 5. Advanced/Metastatic HER2-Negative—Later-Line Treatment

As mentioned above, the earliest approvals for ICI therapy in advanced gastroesophageal malignancies were as later-line therapy in gastric and GEJ adenocarcinomas, with accelerated approval afforded by the FDA for single-agent pembrolizumab in this setting based on KEYNOTE-059 [36], with this approval later withdrawn when confirmatory studies did not demonstrate clinically meaningful improvements in OS for PD-L1 positive gastric and GEJ adenocarcinoma for single-agent ICI therapy. As combined chemotherapy and ICI therapy has become an established standard of care for those with PD-L1 positive/high (or mismatch repair deficient) advanced gastric and GEJ adenocarcinoma in the first-line setting, there have been no further advances for ICI therapy in the later-line settings at the present time.

In comparison, for those with advanced ESCC who did not receive ICI therapy in the first-line setting, single-agent immunotherapy represents a promising option for those requiring second-line systemic therapy and beyond. KEYNOTE-180, a phase II global trial, enrolled 121 patients with advanced ESCC and esophageal or Siewert type 1 GEJ adenocarcinoma who had received at least two prior lines of systemic therapy [53]. ORR for the 63 patients with ESCC was 14.3% (95% CI, 6.7%–25.4%), but just 5.2% (95% CI, 1.1%–14.4%) in the 58 patients with adenocarcinoma histology. Furthermore, for those with PD-L1 CPS ≥ 10 who represented 48% of the total cohort, ORR was 13.8% (95% CI, 6.1%–25.4%) but just 6.3% (95% CI, 1.8%–15.5%) for those who were PD-L1 CPS <10. Expanding on this, KEYNOTE-181 was a phase III trial which randomized N = 628 patients with advanced esophageal adenocarcinoma or ESCC with one previous line of treatment to either pembrolizumab 200 mg every 3 weeks for up to 2 years or investigators’ choice of chemotherapy (paclitaxel, docetaxel or irinotecan) [54]. Almost 64% of patients had ESCC histology, and approximately 37% had PD-L1 CPS ≥ 10. Final analysis was undertaken 16 months after the last randomization, at which median OS for those with PD-L1 CPS ≥ 10 was 9.3 months in the pembrolizumab arm (95% CI, 6.6 to 12.5 months) and 6.7 months for those who received chemotherapy (95% CI, 5.1 to 8.2 months). The co-primary end point of OS in patients with ESCC was not met. Grade 3 or higher AEs occurred in 40.9% of those receiving chemotherapy and 18% of those in the pembrolizumab arm, suggesting this is a more tolerable option for those with previously treated disease. Resulting from KEYNOTE-180 and KEYNOTE-181, the FDA approved single-agent pembrolizumab for ICI naïve advanced ESCC with PD-L1 CPS ≥ 10. The EMA has not afforded approval in this setting.

ATTRACTION-3 soon delivered nivolumab as an alternative option in this setting. A global phase III trial, it randomized N = 419 patients with advanced ESCC previously treated with one line of systemic fluoropyrimidine and platinum-based chemotherapy to either single-agent nivolumab or chemotherapy (paclitaxel or docetaxel) [55]. 96% of patients were Asian, and 48% demonstrated PD-L1 expression ≥ 1%, 35% PD-L1 expression ≥ 5% and 30% PD-L1 expression ≥10%. At minimum follow-up of 17.6 months, median OS in the nivolumab arm was 10.9 months and 8.4 months for chemotherapy (HR 0.77, 95% CI 0.62–0.96, *p* = 0.019). Again, the single-agent immunotherapy was better tolerated than chemotherapy with grade 3–4 AEs documented in 10% of those receiving nivolumab but 23% of the chemotherapy arm. Although those with PD-L1 ≥ 1% had a 15% greater reduction in the risk of death versus those with PD-L1 < 1%, the OS benefit for all outlined above was satisfactory for both the FDA and the EMA to approve nivolumab as monotherapy regardless of PD-L1 status in the second-line treatment setting following fluoropyrimidine and platinum chemotherapy.

More recently, single-agent tislelizumab has been approved by the EMA in the second-line disease setting in ESCC, based on the results of RATIONALE-302, although FDA approval has not yet been granted [56]. N = 512 patients with advanced ESCC, previously treated with chemotherapy in the metastatic/advanced setting, were randomized to either tislelizumab or investigators’ choice of chemotherapy, either paclitaxel, docetaxel or irinotecan. The population was almost 80% Asian and, differing from other studies described here, used a PD-L1 tumor area positivity (TAP) score. The immunotherapy arm demonstrated a statistically significant improvement in median OS at 8.6 months versus 6.3 months with chemotherapy alone [HR 0.70 (95% CI 0.57–0.85); *p* = 0.0001]. For those with PD-L1 TAP score of ≥10%, median OS was 10.3 months with tislelizumab and 6.8 months for chemotherapy alone [HR, 0.54 (95% CI 0.36–0.79); *p* = 0.0006]. These trials of significance in the second and later line treatment of GEC are enumerated in Table 3. 

## 6. Advanced/Metastatic HER2-Positive Gastric/GEJ Cancer

Amplification or overexpression of human epidermal growth factor receptor 2 (HER2/ERBB2) is present in about 20% of advanced gastric and GEJ adenocarcinomas [3,8] with marginally lower levels of expression seen in esophageal adenocarcinomas [57]. Over a decade ago, the seminal ToGA trial established the addition of trastuzumab to chemotherapy as a new standard of care in the first-line treatment of advanced HER2-positive gastric or GEJ cancer [58]. This was the first non-chemotherapy agent to demonstrate statistically significant overall survival benefit in the HER2-positive cohort; however, further progress has been slow. KEYNOTE-811 introduced ICI therapy to the treatment paradigm in this setting, bringing it line with the HER2-negative population [47]. Results of the first interim analysis were published in 2021 for an initial N = 434 patients randomized to either pembrolizumab or placebo in combination with trastuzumab and investigators’ choice of chemotherapy (5-fluorouracil with cisplatin or capecitabine with oxaliplatin). In the intention-to-treat population, about 84% demonstrated PD-L1 CPS ≥ 1 and approximately 80% were HER-2 immunohistochemistry (IHC) 3+ with the remaining roughly 20% IHC 2+. Of note, ToGA defined HER2 positive by fluorescence in situ hybridization (FISH) or 3+ by immunohistochemistry (IHC) [58]. ORRs in the pembrolizumab arm were 74.4% (95% CI, 66.2–81.6) and 51.9% for placebo (95% CI, 43–60.7), consistent with a 22.7% improvement in ORR with the addition of ICI (95% CI, 11.2–33.7; *p* = 0.00006). The pembrolizumab arm also demonstrated a complete response rate of 11.3% versus 3.1% for those in the placebo group. The greater difference in ORR between those with PD-L1 CPS ≥ 1 and those <1, despite the 95% confidence intervals (CIs) overlapping for these subgroups, looks to be the driving factor in the EMA approving the triplet regimen in the first-line treatment setting only for those with PD-L1 CPS ≥1, although the FDA has approved it for all patients regardless of PD-L1 status. Further updated results were presented at ESMO 2023, with final PFS and interim OS results for N = 698 patients at median follow-up of 38.5 months, with dual primary endpoints being OS and PFS [48]. For 85% in the pembrolizumab arm and 86% in the placebo arm, the chemotherapy choice was CAPOX (capecitabine and oxaliplatin), with the remainder receiving FP (5-fluorouracil and cisplatin). PFS at the second interim analysis at 28.4 months was 10 months in the immunotherapy arm and 8.1 months with placebo [HR 0.72 (0.60–0.87); *p* = 0.0002] for all-comers and 10.8 months versus 7.2 months [HR 0.70 (0.58–0.85)] for those with PD-L1 CPS ≥ 1. Similar results were seen at the third interim analysis at 38.5 months; however, these did not meet prespecified criteria for significance and are proceeding to final analysis. Greatest benefit was seen for the those with PD-L1 CPS ≥ 1 with HR 0.71 (0.59–0.86), whereas for those with PD-L1 CPS < 1, HR was 1.03 (0.65–1.64) with potential for harm. Overall survival data continue to mature; however, at the third interim analysis, median OS with pembrolizumab was 20 months in comparison with 16.8 months in the immunotherapy arm in the full population [HR 0.84 (0.74–1.01)], and for those with PD-L1 CPS ≥ 1, it was 20 months with pembrolizumab and 15.7 months with placebo [HR 0.81 (0.67–0.98)]. Updated ORRs were also presented, at 73% with the addition of immunotherapy and 60% in the placebo arm. Grade 3–4 adverse events occurred at 58% with pembrolizumab and 50% for placebo, with the addition of immunotherapy appearing quite well tolerated. HER2-positive gastric/GEJ cancers have been recognized as having a different biology to HER-2 negative disease, with greater PD-L1 expression, higher tumor mutational burden and more tumor infiltrating lymphocytes, and therefore greater immunogenic potential [59]. Previous phase III trials combining anti-PD-1 therapy with chemotherapy in the first-line setting in HER2-negative gastric and GEJ cancers recorded ORRs ranging up to about 60% [38,43], although the almost 15% improvement in ORR to 73% with the addition of trastuzumab suggests there may potentially be a positive interaction between the anti-HER2 agent and ICI therapy and prompts continued investigation in this area.

Focusing on the same cohort, HERIZON-GEA-01 is currently recruiting [49]. A phase III trial, it proposes to randomize previously untreated HER2-postive gastroesophageal adenocarcinoma to either Zanidatamab, a novel bispecific HER2 monoclonal antibody, with chemotherapy (CAPOX or 5-fluorouracil with cisplatin) and tislelizumab, a novel anti-PD-1 antibody. Earlier open-label phase 1b/2 data with the triplet combination was presented at ASCO 2022, with promising results. In a cohort of 33 patients, an ORR of 75.8% (95% CI: 57.7%–88.9%) was reported, including a single patient with a complete response, in addition to a 100% disease control rate (DCR) (95% CI: 89.4%–100%) [60]. However, at time of publication, the only approved regimen combining ICI therapy with anti-HER2 targeted therapy in the HER2+ positive setting is pembrolizumab with trastuzumab and chemotherapy.

## 7. Looking Forward—The Future of Immunotherapy in Gastroesophageal Cancer

As outlined above, over the past decade immunotherapy has firmly established its place as a standard of care treatment across multiple settings in GEC; however, there is considerable scope for this role to be finessed and optimized. While a number of ongoing clinical trials have been highlighted, many more are due to read out in the near future. There is growing understanding of the role angiogenic drivers can play in suppressing the immune microenvironment and thereby limiting the efficacy of immunotherapeutic agents [61]. The LEAP series of trials proposes to synergize the effect of anti-angiogenic targeted agents with ICI therapy given promising pre-clinical anti-tumor activity seen with the combination [50,62,63]. Lenvatinib is a multi-kinase tyrosine inhibitor targeting vascular endothelial growth factor receptor (VEGFR) 1, 2 and 3, fibroblast growth factor receptor (FGFR) 1, 2 and 3 and platelet-derived growth factor receptor (PDGFR alpha), proto-oncogenes c-KIT and RET. LEAP-015 is a two-part phase III trial assessing lenvatinib with pembrolizumab with induction chemotherapy (CAPOX or FOLFOX) followed by maintenance pembrolizumab with lenvatinib in advanced HER2-negative gastroesophageal adenocarcinoma in the first-line setting [64]. Safety run-in data was presented at the 2023 ASCO GI symposium [50]. N = 15 patients received at least one dose of the triplet therapy by median time to data cutoff of 7 months and with an ORR of 75% and a DCR of 93% reported. Treatment-related AEs were seen in 93% of patients, with 53% experiencing grade 3 or 4 AEs. Part 2 of the trial continues to accrue with the combination promising potentially impressive results.

Moving away from immune-adjacent approaches, there are a host of novel immune-based therapies under investigation both as stand-alone agents and in combination with existing anti-PD-L1 ICIs. PD-L1 and cytotoxic T-lymphocyte-associated protein 4 (CTLA-4) represent the two main immune checkpoint receptors currently targeted with current immunotherapy, by limiting tumoral methods of immune escape. However, T cell immunoreceptor with immunoglobulin and ITIM domain (TIGIT) represents a new immune receptor target, as the TIGIT pathway is a regulator of T-cell and NKC (natural killer cell) recognition of tumor cells, playing a role in both the adaptive and immune response against them [65]. Gastric cancers have been shown to utilize this pathway to limit anti-tumor immune response [66], and high TIGIT expression has been suggested to correlate with poorer prognosis [67]. Multiple different anti-TIGIT monoclonal antibodies are under investigation across a host of different malignancies; however, monotherapy with these agents has demonstrated limited ORRs to date and therefore focus has shifted to combination therapy with over 60 different clinical trials incorporating anti-TIGIT therapy active in this area or recruiting at the time of going to publication. This includes STAR-221, a phase III trial looking at domvanalimab (anti-TIGIT) with zimberelimab (anti-PD1) and chemotherapy versus nivolumab and chemotherapy in the first-line treatment of gastroesophageal adenocarcinoma [51]. Confidence in this combination was buoyed by the ARC-7 trial, looking at domvanalimab and zimberelimab with or without etrumadenant (an antagonist of receptors expressed on immune cells to reduce potentially immunosuppressive adenosine in the extracellular domain) in comparison with zimberelimab alone in advanced NSCLC (non-small cell lung cancer) with PD-L1 TPS ≥ 50%, where ORR was 40% with the combination versus 27% with anti-PD-1 therapy alone, and PFS of 12 months versus 5.4 months was seen [68]. Whether anti-TIGIT therapy will establish a role in gastroesophageal malignancies is yet to be determined.

Similarly, lymphocyte activation gene 3 (LAG-3) is another immune checkpoint inhibitory receptor regulating T cell activation, and is active in gastric cancers, although its exact mechanisms in this is as yet not fully understood [69,70]. Combined anti-LAG-3 therapy with relatlimab and anti-PD-1 or anti-PD-L1 therapy has shown promising clinical activity, and has been approved by the FDA and the EMA (with nivolumab) in the management of advanced melanoma on the basis of RELATIVITY-047, where it proved a well-tolerated combination [71]. RELATIVITY-060 is an open-label phase II trial looking at relatlimab with nivolumab and chemotherapy versus nivolumab and chemotherapy in gastric and GEJ adenocarcinoma. This trial has completed enrollment and results are expected in the near future [52].

A final novel immune-based therapeutic approach with potential in the field of gastroesophageal malignancies is Chimeric antigen receptor T-cell (CAR) therapy. A CAR is a manufactured receptor introduced to T cells using viral vectors, and allows the T cell to identify and target certain malignancy-related antigens [72]. The CAR incorporates an extracellular domain which serves to identify the antigens in addition to transmembrane and intracellular signaling domains allowing functionality independent of major histocompatibility molecules. CAR-T cell therapy use is firmly established in a number of hematological malignancies including myeloma, non-Hodgkins lymphoma and acute leukemias, but it has yet to gain a solid foothold in solid organ malignancies. Claudin 18.2 is a normally expressed transmembrane protein in gastric epithelium with expression maintained with malignant transformation [73,74] and is under investigation as a potential target in a number of malignancies including gastric cancer. Very early phase I data have been presented utilizing CAR-T Cells engineered to target claudin 18.2 in a cohort of 37 patients with claudin-expressing GI malignancies, 28 of whom had gastric or GEJ cancers [75]. Almost half of the gastric and GEJ patients had received previous ICI therapy with anti-PD-1 or anti-PD-L1, and an ORR of 57% was seen in this cohort. OS at 6 months was 81.2%, and a 6-month duration of response rate was 53.3%, which raises questions about the durability of response in what would have been a carefully selected cohort. All patients recorded ≥ grade 3 hematological toxicity, and although 94.6% experienced cytokine release syndrome (CRS), no CRS or neurotoxicity ≥ grade 3 was seen. CRS represented a toxicity carrying considerable morbidity and mortality in earlier CAR-T cell therapy trials, and although management of this looks to have been refined by experience gained from its use in other disease settings, later-phase data to support the use of CAR-T Cell therapy in GEC is still pending, and the excessive cost associated with this type of individualized therapy must also be considered.

## 8. Conclusions

GEC represents a broad constellation of histological and molecular subtypes with varying inherent immunogenicity and remains a considerable challenge in terms of the associated morbidity and mortality. Enormous advances have been made over the past decade, establishing immunotherapeutics as a standard of care across a host of settings within the GEC complex, including in both early and advanced disease and in those that are HER2-positive. Despite this, challenges remain in terms of its optimal application across these settings, in particular regarding choice of agent and related biomarker testing. Results of active trials are eagerly awaited, with further advancements to be made both with currently approved anti-PD-L1 and anti-CTLA-4 agents in addition to novel immune-based therapies.

## Figures and Tables

**Table 1:** Peri-operative setting.

	Histology/Setting	Phase/ Region	Arms	Results	Approval
Checkmate 577 [22]	Resected esophageal or GEJ cancer post neoadjuvant chemoradiotherapy with residual pathological disease 71% adenocarcinoma 29% SCC	Phase III Global	Randomized 2:1 A. Nivolumab 240 mg q2/52 for 16/52 then 480 mg q4/52 for max 1 year B. Placebo	Primary Endpoint DFS– mDFS (median disease-free survival): A.22.4 months B.11 months (HR, 0.69; 96.4% CI, 0.56 to 0.86; *p* < 0.001	EMA FDA
EORTC 1707 Vestige [23]	Gastroesophageal adenocarcinoma with lymph node positive or R1 resection post neoadjuvant chemotherapy	Open label randomized Phase II Europe	1:1 A. Chemotherapy as per pre-op regimen B. Nivolumab 3 mg/kg q2/52 + Ipilimumab 1 mg/kg q6/52 × 1 year	mDFS: A. 23.3 months (95% CI 11.8–not reached) B. 11.9 months [8.4–16.8; HR 1.80 (95% CI 1.09–2.98) *p* = 0.02] mOS (median overall survival): A. Not reached B. 25.1 months [95% CI 18.6—not reached (NR); HR 1.79, 95% CI 0.89–3.59; *p* = 0.1.]	N/A (not applicable)
GERCOR NEONIPIGA [5]	Locally advanced resectable dMMR/MSI-H gastric/GEJ Adenocarcinoma T2–T4/N0 or N+/M0	Phase II France	All received neoadjuvant nivolumab 240 mg q2/52 × 6 doses and ipilimumab 1 mg/kg q6/52 × 2 doses then 9 cycles adjuvant nivolumab 480 mg q4/52	N = 32 N = 1—M1 disease at inclusion so not surgical candidate 2× declined surgery All 3 had complete radiological and endoscopic response N = 29 proceeded to surgery Of these— pCR—58.6%	N/A
KEYNOTE-585 [26,27]	Previously untreated, localized, resectable gastric/GEJ andeocarcinoma	Phase III Global multicenter	1:1 A. Neoadjuvant pembrolizumab 200 mg q3/52 with chemotherapy (FP, XP or FLOT) × 3 cycles then adjuvantly × 3 cycles followed by 11 cycles maintenance pembrolizumab B. Placebo + chemotherapy neoadjuvantly then adjuvantly before maintenance placebo	A. pCR 12.9% [95% CI, 9.8–16.6]) B. pCR 2% [95% CI, 0.9–3.9]); Δ (10.9% [95% CI, 7.5–14.8]; *p* < 0.00001) A. median EFS 44.4 months B. 25.3 months (HR 0.81; 95% CI, 0.67–0.99; *p* = 0.0198) – EFS not statistically significant	N/A
MATTERHORN [30,31]	(>T2 N0-3 M0/T0-4 N1-3 M0Resectable Gastric/GEJ adenocarcinoma	Phase III Global	1:1 A. Durvalumab 1500 mg q4/52 with FLOT q2/52 days 1 and 15 for 4 cycles then adjuvant durvalumab B. Placebo + FLOT neoadjuvant and adjuvant placebo	A. pCR 19% B. pCR 7% (12% difference; odds ratio [OR], 3.08; *p* < 0.00001). A. Combined pCR/near-pCRrate 27% B. 14% A. Downstaging to pT0—21% B. Downstaging to pT0 10% A. Downstaging to pN0—47% B. Downstaging to pN0 33%.	N/A
DANTE [32,33]	Resectable gastric or GEJ adenocarcinoma	Phase IIb Germany/Switzerland	A. FLOT + Atezolizumab 840 mg q2/52 × 4 cycles neoadjuvantly and 4 cycles adjuvant then atezolizumab maintenance × 8 cycles q3/52 B. FLOT + placebo	pT0 result: A. 23% B. 15% pN0 result: A. 68% B. 54% Regression—central assessment with PDL1 CPS ≥ 10: Tumor Regression Grade (TRG) 1a A. 46% B. 24% TRG1a/b- 71% vs. 47%	N/A

FP = Cisplatin plus 5-fluorouracil; XP = Cisplatin plus Capecitabine; FLOT = Docetaxel, Oxaliplatin, 5-fluourouracil.

**Table 2:** Advanced disease—first-line treatment.

	Histology/Setting	Phase/ Region	Arms	Results	Approval
KEYNOTE-590 [39,40]	Advanced esophageal and Siewert type 1 gastroesophageal junction cancer regardless of PD-L1 status	Global Phase III	Randomized 1:1 A. Pembrolizumab 200 mg q3/52 + chemotherapy (FP) B. Placebo + chemotherapy	ESCC + PD-LS ≥ 10: A. mOS 13.9 months B. 8.8 months [HR 0.57 (95% CI 0.43–0.75); *p* < 0.0001]. ESCC: A. 12.6 months B. 9.8 months [HR 0.72 (0.60–0.88); *p* = 0.0006] PD-LS ≥ 10: A. 13.5 months B. 9.4 months [HR 0.62 [0.49–0.78); *p* < 0.0001]. All-comers: A. 12.4 months B. 9.8 months [HR 0.73 (0.62–0.86); *p* < 0.0001].	FDA approval for ESCC, EAC + Siewert Class 1 GEJC. EMA approval for ESCC, EAC + Siewert Class 1 GEJC with PD-L1 CPS ≥ 10
CHECKMATE 649 [41,43]	Gastric, GEJ and esophageal adnocarcinoma (Enrollment regardless of PD-L1 expression, but during enrollment primary population amended to those with PD-L1 CPS **≥** 5)	Global Phase III	1:1:1 A. Nivolumab (360 mg q3/52 or 240 mg q4/52) + chemotherapy (XELOX q3/52 or FOLFOX q2/52) B. Ipilimumab and nivolumab C. Chemotherapy alone (later randomized 1:1 after arm B closed)	All-comers: A. mOS 13.8 months C. 11.6 months [HR 0.80 (99.3% CI 0.68–0.94); *p* < 0.0001]. mOS PD-L1 CPS ≥ 1: A. mOS 14 months C. 11.3 months [HR 0.77 (99.3% CI 0.64–0.92); *p* < 0.0001] PD-L1 CPS ≥ 5: A. mOS 14.4 months C. 11.1 months [HR 0.71 (98.4% CI 0.59–0.86); *p* < 0.0001]. All-comers: B. mOS 11.7 months C. 11.8 months [HR 0.91 (96.5% CI 0.77–1.07); *p* value not tested] PD-L1 CPS ≥ 5: B. mOS 11.2 months C. 11.6 months [HR 0.89 (96.5% CI 0.71–1.10), *p* = 0.2302]	FDA approval for nivolumab + chemotherapy regardless of PD-L1 expression EMA for nivolumab + chemotherapy if PD-L1 CPS ≥ 5.
CHECKMATE 648 [44]	Advanced esophageal SCC regardless of PD-L1 expression	Global Open-label Phase III	1:1:1 A. Nivolumab 240 mg q2/52 + FP B. Nivolumab 3 mg/kg q2/52 + ipilimumab 1 mg/kg q6/52 C. Chemotherapy alone	PD-L1 ≥ 1%: A. mOS 15.4 months C. 9.1 months [HR 0.54 (99.5% CI 0.37 to 0.80); *p* < 0.001] B. mOS 13.7 months C. 9.1 months [HR 0.64 (98.6% CI, 0.46 to 0.90); *p* = 0.001] Overall population: A. mOS 13.2 months C. 10.7 months [HR 0.74 (99.1% CI, 0.58 to 0.96) *p* = 0.002]. B. mOS 12.7 months C. 10.7 months [HR 0.78 (98.2% CI, 0.62 to 0.98); *p* = 0.01].	FDA: Nivolumab + 5-Fu and platinum containing chemotherapy and nivolumab + ipilimumab 1st line ESCC regardless of PD-L1 status EMA: Nivolumab + 5-Fu and platinum containing chemotherapy and Nivolumab + ipilimumab 1st line ESCC with PD-L1 ≥ 1%
KEYNOTE-859 [45]	Advanced Gastric/GEJ Adenocarcinoma	Global Phase III	1:1 A. Pembrolizumab 200 mg q3.52 + chemotherapy (FP or CAPOX) B. Placebo + chemotherapy	All-comers: A. mOS 12.9 months B. 11.5 months [HR 0.78, (95% CI 0.70–0.87) *p* < 0.0001] PD-L1 CPS ≥ 1: A. mOS 13 months B. 11.4 months [HR 0.74 (95% CI 0.65–0.84) *p* < 0.0001] PD-L1 CPS ≥ 10: A. mOS 15.7 months B. 11.8 months [HR 0.65 (95% CI 0.53–0.79) *p* < 0.0001)	FDA approved regardless of PD-L1 status EMA approval for PD-L1 CPS ≥ 1.
RATIONALE 306 [46]	Advanced ESCC regardless of PD-L1 expression First-Line	Global Phase III	1:1 A. Tislelizumab + chemotherapy (cisplatin or oxaliplatin + capecitabine or fluoropyrimidine or paclitaxel) B. Chemotherapy alone	mOS: A. 17.2 months (95% CI 15.8–20.1) B. 10.6 months [HR 0.66 (95% CI 0.54–0.80); *p* < 0.0001]	FDA: under review EMA: Not approved (tislelizumab approved in 2nd line setting as single agent)
KEYNOTE-811 [47,48]	HER-2 positive (IHC 2+ and 3) gastric and GEJ adenocarcinoma	Global Phase III	1:1 A. Pembrolizumab + Trastuzumab + chemotherapy (FP or CAPOX) B. Placebo + Trastuzumab + chemotherapy	(At 2nd interim analysis) A. mPFS 10 months (95% CI 8.6–11.7) B. mPFS 8.1 months [HR 0.72 (95% CI 0.60–0.87); *p* = 0.0002] A. mOS 20 months B. mOS 16.9 months [HR 0.87 (0.72–1.06); *p* = 0.084]	FDA approved all-comers EMA approved PD-L1 CPS ≥ 1
HERIZON-GEA-01 [49]	Advanced HER2+-positive gastric/GEJ and esophageal adenocarcinoma (IHC3+ or IHC2+/ISH+)	Global Phase III	1:1:1 A. Trastuzumab + chemotherapy (CAPOX or FP) B. Zanidatamab (novel bispecific anti-Her2 antibody) + chemotherapy C. Zanidatamab + chemotherapy + tislelizumab	-	Recruiting
LEAP-015 [50]	Advanced gastroesophageal adenocarcinoma (first line)	Global Phase III	1:1 A. Pembrolizumab 400 mg q6/52 + Lenvatinib 8 mg QDS + chemotherapy (CAPOX or mFOLFOX6) then consolidation pembrolizumab 400 mg q6/52 with lenvatinib 20 mg QDS B. Chemotherapy alone	Part 1 reported: Safety run in: non-randomized. All patients received pembrolizumab + Lenvatinib + chemotherapy. N = 15 patients ORR 73% (95% CI; 45–92) DCR 93% (95% CI; 68–100)	Randomized part 2 ongoing
STAR-221 [51]	Advanced gastric, GEJ and esophageal adenocarcinoma	Global Phase III	1:1 A. Domvanalimab + Zimberelimab + chemotherapy (FOLFOX or CAPOX) B. Nivolumab + chemotherapy	-	Recruiting
CA224-060 [52]	Gastric/GEJ Adenocarcinoma	Global Phase II	A. Relatlimab + nivolumab + chemotherapy (oxaliplatin based) B. Nivolumab + chemotherapy	-	Active, not recruiting

FP = Cisplatin plus 5-fluorouracil; XELOX = Xeloda (Capecitabine) plus Oxaliplatin; FOLFOX = 5-fluourouracil, Folinic Acid, Oxaliplatin (mFOLFOX = modified FOLFOX).

**Table 3:** Advanced disease—second-/later-line treatment.

	Histology	Phase/Region	Arms	Results	Approval
KEYNOTE-059 [36]	Advanced gastric/GEJ adenocarcinoma	Phase II Global Open-label Non-randomized	Pembrolizumab 200 mg q3/52 up to 35 cycles	PD-L1 ≥ 1: ORR 15.5% (95% CI; 10.1%–22.4%; 23 of 148 patients) CR 2% (95% CI, 0.4%–5.8%) PD-L1 < 1 ORR 6.4% (95% CI; 2.6%–12.8%) CR 2.8% (95% CI; 0.6%–7.8%).	Accelerated FDA approval in 2017, approval withdrawn in 2021
KEYNOTE-181 [54]	Advanced ESCC and esophageal adenocarcinoma	Global Phase III	A. Pembrolizumab 200 mg q3/52 B. Chemotherapy (paclitaxel, docetaxe, irinotecan)	CPS ≥ 10: A. mOS 9.3 months 6.7 months [HR 0.69 (95% CI, 0.52 to 0.930; *p* = 0.0074]. ESCC: A. mOS 8.2 months B. 7.1 months [HR 0.78 (95% CI, 0.63 to 0.96); *p* = 0.0095] All patients A. 7.1 months B. 7.1 months [HR 0.89 (95% CI, 0.75 to 1.05); *p* = 0.0560].	FDA approved for ICI naïve advanced ESCC with PD-L1 CPS ≥ 10. EMA not approved
ATTRACTION-3 [55]	Advanced ESCC	Global Phase III	A. Nivolumab 240 mg q2/52 B. Chemotherapy (paclitaxel or docetaxel)	A. mOS 10.9 months (95% CI 9.2–13.3) B. 8.4 months [HR 0.77, (95% CI 0.62–0.96) *p* = 0.019]	FDA and EMA approved
RATIONALE-302 [56]	Advanced ESCC post first-line chemotherapy	Global Phase III	A. Tislelizumab B. Chemotherapy alone (docetaxel, paclitaxel or irinotecan)	All-comers: A. mOS 8.6 months B. 6.3 months [HR 0.70 (95% CI, 0.57–0.85); *p* = 0.0001) PD-L1 TAP ≥ 10%: A. mOS 10.3 months B. 6.8 months [HR 0.54 (95% CI 0.36–0.79]; *p* = 0.0006).	Not FDA approved. EMA Approval regardless of PD-L1
